# Modifying Epigenetic Landscapes to Restore Immune Therapeutic Responses in Triple Negative Breast Cancer

**DOI:** 10.3390/cancers18081221

**Published:** 2026-04-12

**Authors:** Nabeelah Almalki, Mercedes Vázquez-Cantú, Riba Thomas, Tinyiko Modikoane, Mansour Alsaleem, Jenny Persson, Emad Rakha, Nigel P. Mongan, Cinzia Allegrucci

**Affiliations:** 1 SVMS and Nottingham Breast Cancer Research Centre, Biodiscovery Institute, University of Nottingham, Nottingham NG7 2RD, UK; nabeelah.almalki1@nottingham.ac.uk (N.A.); mercedes.vazquez_cantu@nottingham.ac.uk (M.V.-C.); riba.thomas@nottingham.ac.uk (R.T.); tinyiko.modikoane@nottingham.ac.uk (T.M.); mansour.alsaleem1@nottingham.ac.uk (M.A.); nigel.mongan@nottingham.ac.uk (N.P.M.); 2 Department of Clinical Laboratory Sciences, College of Applied Medical Sciences, Shaqra University, Shaqraa 11961, Saudi Arabia; 3 Unit of Scientific Research, Applied College, Qassim University, Buraydah 52571, Saudi Arabia; 4 Department of Molecular Biology, Umea University, 901 87 Umea, Sweden; jenny.persson@umu.se; 5 School of Medicine, University of Nottingham, Nottingham NG7 2RD, UK; emad.rakha@nottingham.ac.uk; 6 Nottingham University NHS Trust, Nottingham NG7 2UH, UK; 7 Department of Pharmacology, Weill Cornell Medicine, New York, NY 10065, USA

**Keywords:** triple negative breast cancer, epigenetics, immune evasion, immunotherapy, tumor microenvironment, transposons, viral mimicry, epigenetic therapy

## Abstract

Triple-negative breast cancer (TNBC) is an aggressive subtype of breast cancer defined by the absence of estrogen and progesterone receptors, as well as the lack of human epidermal growth factor 2 receptor overexpression. TNBC is associated with early onset, high metastatic potential, therapeutic resistance, and poor clinical outcomes exacerbated by the limited availability of effective targeted therapies. Advances in multi-omics profiling have further stratified TNBC into distinct molecular subtypes, each exhibiting unique genomic, epigenomic, and immune-related features that influence therapeutic responsiveness. This review explores the interplay between TNBC molecular heterogeneity, immune evasion mechanisms, and epigenetic regulation. TNBC demonstrates variable immunogenicity, with tumor-infiltrating lymphocytes serving as important prognostic and predictive biomarkers. However, immune escape commonly occurs through tumor microenvironment remodeling, T-cell exhaustion, cancer stem cell enrichment, and immune checkpoint pathways activation. Although immune checkpoint inhibitors have improved outcomes in selected patients, particularly in combination with chemotherapy, primary and acquired therapeutic resistance remain a significant challenge. Emerging evidence highlights the central role of epigenetic mechanisms in regulating immune-related gene expression and shaping the tumor immune microenvironment. Epigenetic silencing of antigen presentation machinery, interferon signaling pathways, and chemokine expression contributes to immune evasion and immunotherapy resistance. Importantly, pharmacological modulation of epigenetic regulators can restore immune recognition and induce “viral mimicry” through reactivation of endogenous retroelements, thereby enhancing antitumor immunity. Collectively, this review underscores the therapeutic potential of integrating epigenetic therapies with immunotherapy and chemotherapy to overcome immune resistance in TNBC. A deeper understanding of epigenetic-immune interactions may facilitate the development of more precise and effective treatment strategies tailored to TNBC molecular subtypes.

## Simple Summary

Triple negative breast cancer (TNBC) is an aggressive form of breast cancer that does not express estrogen and progesterone receptors, together with a lack of human epidermal growth factor 2 receptor overexpression. As a result, many conventional targeted treatments are ineffective. TNBC is not a single entity but comprises multiple subtypes with distinct genetic and molecular characteristics that influence treatment response and clinical outcome. Although TNBC can stimulate an immune response, many tumors develop mechanisms to evade immune detection and destruction. These include modification of the tumor environment, suppression of immune cell activity, and activation of immune checkpoint pathways that impair T cell function. Emerging evidence indicates that epigenetic alterations, chemical modifications that regulate gene expression without changing the DNA sequence, play an important role in facilitating immune escape. Therapeutic strategies targeting these epigenetic mechanisms may restore anti-tumor immunity and improve the effectiveness of existing treatments, offering promising avenues to improve outcomes for patients with TNBC.

## 1. Introduction

Triple Negative Breast Cancer (TNBC) accounts for approximately 15–20% of all breast cancer cases and is defined by the lack of expression of the estrogen receptor, progesterone receptor, and lack of human epidermal growth factor 2 receptor overexpression. TNBC is frequently associated with early disease onset and aggressive clinical behavior [1]. The aggressiveness of these tumors is mainly attributed to high invasiveness, increased metastatic potential, and treatment resistance. Consequently, TNBC presents an increased risk of distant recurrence, with shorter progression-free survival and overall survival. The five-year overall survival is approximately 77% in early-stage disease, but declined dramatically to around 12% in advanced or metastatic settings [2]. Therapeutic options remain limited, as the absence of receptor expression precludes the use of established targeted endocrine and HER2-directed therapies. As a result, TNBC is primarily managed with a combination of surgery, radiotherapy, and chemotherapy [3].

TNBC molecular heterogeneity also limits the therapeutic efficacy. TNBC is more heterogeneous than other BC subtypes, and is currently classified based on multi-omics profiling, incorporating mutations, copy number alterations, gene expression, DNA methylation, and proteomic signatures into four subtypes: basal-like 1 (BL1), basal-like 2 (BL2), luminal androgen receptor (LAR), and mesenchymal (M) [4]. This analysis identifies genomic and epigenomic differences within TNBC subtypes, highlighting unique molecular characteristics and therapeutic vulnerabilities of each subtype.

Genomic and epigenomic heterogeneity among TNBC subtypes suggests distinct cells of origin and divergent molecular pathways. The BL1 subtype has been associated with bipotent progenitor cells, suggesting that basal-type breast tumors may arise from the luminal epithelial lineage rather than basal cells [5]. In contrast, BL2 and M-subtypes demonstrate features consistent with myoepithelial/basal cell differentiation. However, the global DNA hypomethylation observed in the M-subtype may reflect tumor dedifferentiation rather than a distinct lineage. Similarities in copy number alteration and mutational profiles between M and BL1 subtypes are similar, suggesting that M-subtype tumors may evolve from BL1 precursors [4]. BL tumors are characterized by high genomic instability and mutational burden. Due to their high expression of DNA damage response genes, these tumors tend to have a better response to chemotherapy and radiotherapy. Most cancers arising in patients carrying BRCA1 mutations are basal-like, making them responsive to platinum-based chemotherapy and poly ADP ribose polymerase (PARP) inhibitors, which target cells unable to undergo DNA repair by homologous recombination [6]. Abnormal activation of other signaling pathways also characterizes BL2 tumors, and potential therapeutic agents include growth factor inhibitors or mTOR inhibitors. BL tumors have been shown to express high levels of EGFR, and its inhibition improves response to chemotherapy in pre-clinical models [7]. M-type tumors are also characterized by high mutational rates, but they also show expression of stemness and epithelial-to-mesenchymal transition genes (EMT). These tumors are characterized by high activation of differentiation pathways, cell migration-related signaling pathways, and extracellular matrix–receptor interaction pathways. M-subtype tumors might be treated with mTOR inhibitors, but they are more difficult to treat and targeted therapies are limited for this tumor subtype. Finally, the LAR tumors are characterized by highly activated hormone-related signaling pathways, including PI3K signaling. Therefore, they respond to AR antagonists and PI3K/AKT/mTOR inhibitors [4,8].

Classification of tumors based on immune-related gene signatures has recently opened new avenues for the use of immunotherapy for TNBC. Although TNBC is more immunogenic than other breast cancer subtypes due to enrichment of tumor neoantigens and high mutational burden, evident differences exist among the different subtypes, which impact patient survival and therapy response, with only a minority of patients benefiting from immunotherapy [9]. These differences are the result of different mechanisms that regulate immune evasion and the complex interaction between TNBC cells and the tumor microenvironment (TME).

## 2. TNBC and Immune Evasion

The immunological landscape of TNBC is shaped by immunoediting mechanisms, which determine the recognition and destruction of cancer cells. During cancer progression, cancer cells are initially eliminated by innate and adaptive immune responses recognizing the aberrant expression of tumor antigens. However, tumor cells acquire molecular changes that allow them to enter an equilibrium phase, which enables escape from the immune response because of a selective growth advantage, ultimately leading to immune evasion [10]. Key to the immune escape is the interaction with the TME and immune cells, which are recruited during the immune response to infiltrate the tumor. TNBC exhibits immunogenic properties, and the presence of tumor-infiltrating lymphocytes (TILs), as effectors of immune response, serves as a predictive and prognostic marker. Indeed, the presence of TILs significantly correlates with good prognosis and response to immunotherapies targeting the interaction between antigens expressed on T lymphocytes and cancer cells [11]. Moreover, the distinct presence of immune cells, their spatial distribution, and the expression of checkpoint antigens can be used to stratify TNBC and predict prognostic outcomes [12].

Tumors that exhibit infiltration of granzyme B+CD8+ T cells (GzmB+CD8+ T cells) and elevated expression of multiple immune molecules in the stroma and epithelial tumor compartments show an immunoreactive microenvironment and result in a good outcome. Immunoreactive tumors also present an inflamed environment and high interferon (IFN) signaling, and expression of antigen presentation genes, such as TAP and B2M. They are also infiltrated with proinflammatory macrophages and enriched with an immunomodulatory signature [13]. On the other hand, TNBC with an immune-cold microenvironment shows an absence of CD8+ T cell tumor infiltration or infiltration restricted to the tumor margins. It also shows enrichment of fibrotic foci, elevated expression of the fibrotic stroma marker B7-H4, loss of the MHC-I molecules, high TGFβ signaling, and a mesenchymal subtype signature correlated to poor outcome [12]. Deletions in beta-2-microglobulin (B2M) are more frequent in the immune-cold M-subtype compared to other subtypes, impacting antigen presentation and the efficacy of immune checkpoint inhibitors [4].

Based on their immune landscape, immunotherapy can be used to target immunoreactive tumors using immune checkpoint inhibitors (ICIs) which rely on the interaction of co-inhibitory receptors expressed (PD1, CTLA-4, LAG3) on the surface of T cells and ligands expressed on cancer cells (PD-L1) or antigen presenting cells (CD80/86, MHC) to suppress T cell-mediated immune responses [14]. By inhibiting this checkpoint interaction, immune responses can be successfully re-established against cancer cells [15]. PD-1 inhibitors (Nivolumab, Pembrolizumab, and Cemiplimab), PD-L1 inhibitors (Atezolimumab, Durvalumab, and Avelumab), and CTLA-4 inhibitors (Ipilimumab) are immune checkpoint inhibitors (ICIs) currently investigated for TNBC treatment [16].

However, although all TNBC subtypes show expression of PD-L1, ICIs have shown modest effects on advanced TNBC patients as a monotherapy [11]. Response rates significantly improve when the ICIs are used in combination with chemotherapy, especially in tumors expressing PD-L1. To this end, the inhibitor Pembrolizumab is currently approved for use in both early and advanced TNBC in combination with paclitaxel or gemcitabine-carboplatin [17].

Resistance to ICIs results from the effect of the adaptive immune system within tumor cells, which alters the TME during cancer progression and confers resistance to immune responses. The acquisition of adaptive resistance can occur through the loss of antigens, the disruption of cell signaling cascades, and/or extended stimulation of T cells, leading to their exhaustion. This ultimately leads to the suppression of cytokine release and a subsequent reduction in immunological efficacy [18]. Therefore, the prognostic value of TILs in TNBC is related to the overall balance of the different types of infiltrating lymphocytes with either a positive or negative antitumor effect. Immune cells in the TME include regulatory T cells (Tregs), myeloid-derived suppressor cells (MDSCs), natural killer cells, macrophages, dendritic cells, tumor-associated macrophages (TAMs), tumor-associated neutrophils, and cytokines such as TGF-β, which induce T cell exhaustion. Therefore, even if present in the tumors, exhausted effector T cells progressively lose the ability to kill cancer cells [19]. Among immunosuppressive T cells, Tregs are of particular interest as they are abundant in TNBC and induce an immune evasive microenvironment, inhibiting CD8+ and CD4+ T-cell activation and correlating with PD-L1 expression in TNBC [11,20]. Importantly, Tregs are involved in cytotoxic T cells exhaustion by preventing excessive immunopathological damage, a process that is triggered by tumor growth, altered nutrient composition and oxygen availability, as well as by the release of cytokines and chemokines in the TME. For instance, it has been shown that IL-10 and IL-35 secreted by Tregs facilitate CD4+ and CD8+ T cell exhaustion by epigenetically activating BLIMP-1-mediated expression of PD-1, LAG3, TIM3, TIGIT, and 2B4 [21]. T cell exhaustion is a gradual and progressive process that occurs because of chronic antigen exposure in the TME. Although initially responsive to the block of immune checkpoint, T cells eventually acquire partial responsiveness. Exhausted CD4+ and CD8+T cells exhibit reduced production of cytokines and proliferation, all contributing to their impaired cytotoxicity. With persistent stimulation, they acquire high expression of inhibitory receptors such as PD-1, TIM-3, CTLA-4, and LAG-3, diminished cytokine production, and impaired metabolism through epigenetic reprogramming [19]. The degree of T cell exhaustion also alters the response to chemotherapy and targeted therapies. Indeed, the T cell-exhausted tumors respond to agents targeting kinases and transcriptional regulators, whereas the tumors with low exhaustion are sensitive to chemotherapy, including 5-FU and docetaxel [22]. This is consistent with the evidence that TNBC with an immunoreactive microenvironment tends to have an improved response to neoadjuvant chemotherapy [23].

Cancer stem cell (CSC) enrichment in TNBC also contributes to T cell exhaustion and tumor immune evasion through intrinsic resistance, such as the expression of ICIs, and by modulating the TME [24]. This critical connection has been clearly demonstrated by the negative correlation between CSCs and the immune landscape in solid tumors, whereby increased cancer stemness has been associated with a reduced presence of CD8+ T cells, NK and B cells, as well as increased polarization of macrophages in the TME [25]. Through a phenomenon of adaptation and sustained plasticity, CSCs show enhanced DNA repair, anti-apoptotic signalling, autophagy, and EMT, which support survival under immune pressure. Both EMT and autophagy reduce susceptibility to T cell-mediated lysis, inhibit cell killing by natural killer cells, and reduce the infiltration of CD4+ TILs [24]. CSCs can also downregulate MHC-I antigen presentation and alter IFN and STAT3/IL-6 pathways, limiting recognition by cytotoxic T cells and promoting the secretion of immunosuppressive cytokines [26,27]. As MHC-I loss can drive NK activity, CSCs bypass this toxic effect by reducing the expression of MICA and MICB, two ligands for the stimulatory NK cell receptor NKG2D [26].

In addition to the effect on NK cells, cytokines and other growth factors secreted by CSCs, such as CCL2, IL-10, TGF-β, and VEGF, induce the differentiation of the immunosuppressive and pro-tumor M2-macrophages. In turn, the induced M2 macrophages sustain the CSC phenotype by secreting IL-10, TGFbeta, and EGF, which enhance stem cell self-renewal, EMT, angiogenesis, and metastatic potential [28].

The recruitment of TAMs, neutrophils, and Tregs is also sustained by mediators such as macrophage migration inhibitory factor (MIF) that reprogram tumor metabolism, potentiating glycolysis and WNT/β-catenin signalling, fostering an immunosuppressive environment, as well as inducing the expression of TIM3, PD-1, and TOX on CD4+T lymphocytes [29]. Therefore, the immune landscape and the CSCs enrichment of TNBC have important effects on T cell exhaustion and implications for immunotherapy.

**Figure 1:**
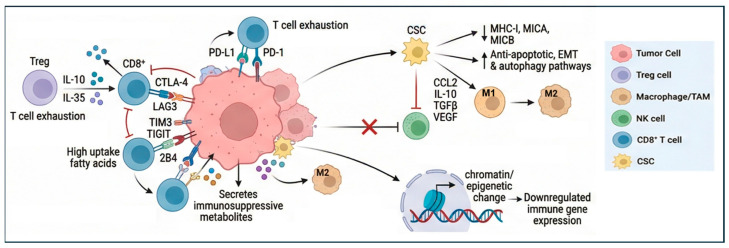
Mechanisms of TNBC immune evasion. Immune-cold tumors are characterized by lack of infiltration CD8+ effector T cells and infiltration of immunosuppressive immune cells within the TME, including Tregs and TAMs. Cytokines and immunosuppressive metabolites, together with cancer stemness and epigenetic mechanisms, contribute to the creation of an environment that induces T cell exhaustion and immune checkpoint pathways, which drive cancer immune evasion.

Different strategies are currently under investigation, including combination therapies, metabolic modulators, and next-generation cell transfer therapy approaches designed to prevent or reverse the immunosuppressive state of the TME [30]. Combination therapies using checkpoint inhibitors are currently being investigated to enhance immune responses. Targeting multiple checkpoints (e.g., PD-1 plus TIM-3, LAG-3, B7-H3, CTLA-4) could provide more effective therapies. Another strategy is exploiting the adoptive cell transfer therapy (ACT) approach, which includes the isolation of autologous immune cells and subsequent reinfusion after their engineering to achieve functional enhancement. ACT strategies include the use of adoptive TILs therapy, T-cell receptor chimeric T-cell (TCR-T) therapy, and chimeric antigen receptor T-cell (CAR-T) therapy. Importantly, TIL therapy in conjunction with PD-1 blockade has shown improved outcomes [31]. CAR-T engineered to express genes that counteract exhaustion, together with chemokines or antibodies blocking PD-1, has recently demonstrated promising pre-clinical benefits [19]. Modulation of metabolism can also prevent the progression of T cell exhaustion. TNBC is characterised by active cancer cell growth, impacting nutrient availability and metabolism. By competing for nutrient availability with T cells, cancer cells can produce immunosuppressive metabolites. These metabolites include amino acids and glucose, which contribute to CD8+T exhaustion. For instance, metabolism of tryptophan and conversion to aromatic hydrocarbon receptor (AhR) suppresses the production of IL2 secreted by T cells and induces the expression of inhibitory receptors, including PD-1, LAG3, and TIM3. Tumour cells also secrete molecules that affect glucose metabolism. By releasing CD155, they inhibit the PIK/AKT pathways and the uptake of glucose into T cells through repression of the glucose uptake receptor GLUT1 [19]. Lipid metabolism also impacts the TNBC immune environment. Alterations in the lipid uptake and storage, as well as fatty acid synthesis and oxidation, affect the function of immune cells. This is particularly evident during TNBC progression, as tumor cells abnormally uptake fatty acids, reducing their availability for immune cells. In turn, this affects the polarization of TAMs to an M2 phenotype. Importantly, PGE2 produced by TAMs upregulates PD-L1 expression, leading to T cell inhibition [32]. Finally, modulation of epigenetic programs is another important strategy for preventing tumor immune escape. Epigenetic modifications of the chromatin can regulate gene expression of both tumor cells and cells of the TME, thus affecting the immune landscape of TNBC. Therefore, we propose that epigenetic dysregulation acts as a functional bridge between intrinsic TNBC molecular subtype and extrinsic immune phenotype. Specifically, subtype-specific epigenetic alterations modulate chromatin accessibility, cytokine networks, antigen presentation machinery, and endogenous retroviral activation, thereby shaping immune infiltration patterns and therapeutic responsiveness. This framework suggests that epigenetic mechanisms may function as primary drivers of immune-cold phenotypes, adaptive mediators of immune escape under therapeutic pressure, or consequences of genomic instability that secondarily remodel the TME (Figure 1).

## 3. Epigenetic Regulation of Immune Responses

### 3.1. Epigenetic Landscape of TNBC

Epigenetic mechanisms contribute to cancer immune escape by regulating the expression of immune-related genes through changes in DNA methylation, as well as histone modifications and RNA interference. TNBC presents a characteristic epigenetic landscape, with altered expression of epigenetic modifiers that modulate the levels of DNA methylation and histone modifications.

DNA methylation involves the transfer of a methyl group to cytosine, occurring at cytosine-guanine dinucleotides (CpG) to control gene expression by repressing transcription factors binding to DNA. This reaction is catalyzed by the DNA methylation enzymes (DNMTs), which methylate cytosines mostly at transposable elements and at gene promoters. This epigenetic signature is disrupted in cancer, leading to genome-wide hypomethylation, loss of silencing at oncogene promoters, and silencing of tumor suppressor genes [33]. In TNBC, the expression of the DNMT enzymes is altered, and this leads to global hypomethylation, alongside promoter hypermethylation of tumor suppressors, driving oncogenesis and immune evasion [34]. Importantly, these aberrant DNA methylation signatures are correlated with poor patient prognosis, as they are associated with resistance to chemotherapy and immunotherapy [35]. DNA methylation levels differ among different TNBC subtypes, and this directly affects their immune environment [34].

A multi-omics analysis of the different TNBC molecular subtypes was conducted by Lehmann et al. [4] demonstrated that LAR tumors display a higher number of differentially hypermethylated CpGs located at the promoter region of genes and within 3 kb from promoters, whereas the M subtype has the most hypomethylated CpGs. BL tumors were found to have intermediate levels of DNA methylation. A more recent genome-wide methylome profiling of TNBC identified two epitype subgroups of basal and non-basal tumors. The non-basal epitype tumors corresponded to the LAR molecular subtype, whereas the basal epitype group was most represented by BL and M molecular subtypes. The non-basal group presented higher levels of methylation compared to the basal group and a lower response to chemotherapy [36]. The two epitypes also showed different levels of immune infiltration, with the basal group presenting higher TIL numbers compared to the non-basal group. Three further subgroups were identified within the basal epitype group, with group 3 (Basal 3) presenting higher lymphocyte infiltration compared to the intermediate group 2 (Basal 2) and low-infiltration group 1 (Basal 1). Basal 1 tumors were heterogeneous and mostly associated with metabolic pathways. On the other hand, Basal 2 tumors were enriched in the M molecular subtype and EMT, TGFβ, and hypoxia signatures. This was consistent with the previous report showing that M molecular subtype tumors exhibit a highly hypomethylated genome and hypermethylation of promoters of antigen presentation genes [4]. Basal 3 tumors were mostly associated with the molecular subtype BL1/BL2 tumors, immune pathways, and showed a good correlation with hypomethylation and high expression of PD-L1 and PD-L2 genes. The basal 3 subgroup also showed the highest number of immune cell infiltration, compared to intermediate levels of the basal 2 group and the low values of the basal 1 group, as demonstrated by TILs infiltration scores and immune response gene expression profiling [36].

Together with DNA methylation, histone modifications also contribute to the epigenetic landscape of TNBC. Histone modifications encompass a range of post-translational biochemical reactions, including methylation, acetylation, phosphorylation, and ubiquitination [37]. These modifications alter the chromatin conformation to allow a state that is permissive (euchromatin) or repressive (heterochromatin) for transcription. Histone acetylation neutralizes positive charges, loosening DNA-histone interactions, leading to euchromatin that is accessible to the transcription machinery and supports active gene expression. This modification is catalyzed by histone acetyltransferase enzymes (HATs) and erased by histone deacetylases (HDACs). Histone methylation encompasses a series of complex modifications that either activate or repress transcription depending on the residue and methylation state. Methylation of histone H3 at lysine 4 (H3K4me3) is an active mark, while H3K9me3, H3K27me3, and H4K20me3 promote condensed heterochromatin and gene silencing. Histone methyltransferases (HMTs) and histone demethylases (KDMs) add and remove histone methylation marks, respectively [38]. A recent multi-omics study revealed the histone modifications profile of TNBC, highlighting a marked decrease in H4K20me3 and H4K16ac, known hallmarks of cancer [39]. It also showed an increase in H4me1/me2, H3K9me3, H3K36m1/me2 and H4 hyper-acetylation, whereas H3K27me3 and H3K79me1/me2 were decreased. Importantly, increased H3K4me2, together with the expression of the methyltransferase enzyme KMT2B, was found to sustain TNBC phenotypes and was negatively associated with overall survival and disease-free survival [40]. Histone demethylases of the lysine-specific demethylase 1 (LSD1/KDM1) or the Jumonji C (JmjC) domain family (KDM2 to KDM8) also play an important role in TNBC. Particularly, the enzymes KDM2B, KDM4C, KDM5B, KDM1A/LSD1, KDM4A, and KDM5A have been shown to drive cancer progression by removing active methyl marks (e.g., H3K4me3, H3K36me2), repressing tumor suppressors, and promoting proliferation, invasion, and immune evasion [41]. Among molecular subtypes, the basal-like demonstrates an enrichment of the histone methyltransferases EZH2, SMYD2, and SETDB1. These enzymes catalyzing the methylation of H3K27, H3K4, and H3K9, respectively, have been shown to play important roles in maintaining the aggressive phenotype of basal-like tumors through regulation of cell proliferation, invasion, and cancer stemness [42].

**Figure 2:**
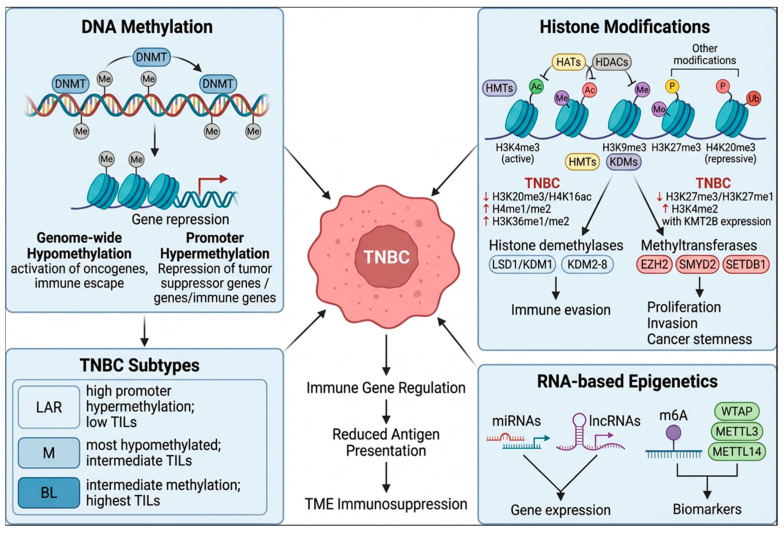
Epigenetic mechanisms, including alterations in DNA methylation, histone modifications, and non-coding RNA regulation, drive immune escape and tumor progression in triple-negative breast cancer by reshaping immune-related gene expression across distinct molecular subtypes.

Together with DNA methylation and histone modifications, other epigenetic mechanisms that regulate gene expression at the post-transcriptional level have been highlighted as playing an important role in TNBC. These include the expression of non-coding RNAs, which contribute to TNBC phenotypes, as well as RNA methylation [43]. Several regulators involved in N6-methyladenosine (m6A) RNA modification have been reported to be significantly dysregulated in TNBC and to function as prognostic biomarkers [44] (Figure 2).

### 3.2. Epigenetic Modifications of Immune Genes

#### 3.2.1. DNA Methylation and Histone Modifications

DNA methylation and histone modifications play a critical role in cancer immune evasion by silencing immune genes, including tumour-associated antigens and those involved in antigen presentation and IFN signalling [34]. For instance, several cancer-testis antigen genes are often methylated in TNBC, thus reducing antitumour immune responses [45]. The expression of MHC class I and II and associated HLA genes is also regulated by DNA methylation, and hypermethylation of HLA promoters also correlates with decreased CD8A expression in TNBC [46]. Methylation of the immunoproteasome gene PSMB9 also drives immune evasion by compromising antigen processing and MHC class I presentation [47]. Hypermethylation of checkpoint receptors, such as PD-L1, reduces the immune recognition of cancer cells by cytotoxic T lymphocytes, promoting immune evasion and resistance to immunotherapies [48]. Indeed, methylation of PD-1 and PD-L1 correlates with immune infiltration in TNBC, particularly the silencing of PD-1, which is associated with macrophage infiltration [49]. The expression of PD-L1 in TNBC is also associated with loss of ZNF62, a transcriptional repressor that represses PD-L1 expression through interaction with the NuRD chromatin remodelling complex [50]. Silencing of the IFN signalling-related genes, such as the Stimulator of Interferon Response cGAMP Interactor 1 (STING1), has also been observed in TNBC. This can lead to a reduction in the secretion of the chemokines CCL5, CXCL10, and CXCL11 and reduced recruitment of immune cells into the tumour microenvironment [51].

Other epigenetically regulated genes have been identified to induce immune evasion in TNBC. The methylation of the transcriptional repressor ZBTB28 induces the expression of CD47 and CD24, two antigens involved in the inhibition of immune response mediated by macrophages [52]. On the other hand, hypomethylation of LGALS2 promotes the polarization and proliferation of the immunosuppressive M2 macrophages [53]. For T cells, methylation of the TOX (Thymocyte Selection-Associated High Mobility Group Box) gene, which encodes a nuclear DNA-binding protein crucial for T lymphocyte development, differentiation, and the regulation of immune cell exhaustion, is critical for promoting the expression of exhaustion-associated gene signatures, with high levels of TOX expression being inversely correlated with the level of the master regulator of T-cell development TCF1 and associated with elevated expression of multiple inhibitor receptors [54]. Therefore, treatment with DNMT inhibitors (DNMTi) can restore the expression of tumor-associated antigens, immune checkpoint receptors, and antigen-presenting genes, such as MHC class I, leading to enhanced T cell-mediated immune responses [55].

Histone modifications cooperate with DNA methylation in regulating the expression of immune genes. In the case of PD-L1, its expression is positively correlated with the activity of the histone deacetylase enzyme HDAC2 through chromatin remodelling and activation of the IFN/JAK pathway, which phosphorylates and activates PD-L1 [56]. Therefore, HDAC2 inhibition increases TILs in TNBC mouse models by downregulating PD-L1 expression [56]. Inhibition of epigenetic readers like the Bromodomain and ExtraTerminal (BET) proteins, which interact with acetylated lysine residues, has also been shown to control the expression of PD-1 in activated T cells and PD-L1 in TNBC cells, with a positive effect in counteracting T cell exhaustion and restoring T cell-mediated cytotoxicity [57].

Other hypomethylated checkpoint receptors, including TIM-3, CTLA-4, and LAG3, present low levels of the repressive histone marks H3K27me2 and H3K9me3 at their promoter regions, which contribute to their expression [58]. By removing histone active marks, the lysine-specific demethylase 1 (LSD1) is responsible for silencing the T cell-attracting chemokines CCL5, CXCL9, and CXCL10 chemokines, as well as PD-L1 expression [59]. In addition, by interacting with histone demethylase KDM5B, LSD1 inhibits the chemokine CCL14, which promotes immune cell activation [56]. Another histone-modifying enzyme, which is critical for the regulation of immune genes, is the histone methyltransferase EZH2, a H3K27 methyltransferase of the polycomb repressor complex 2 (PRC2). EZH2 is upregulated in TNBC, and its activity is involved in T cell exhaustion, with high expression observed in CD8+ T cells expressing PD-1 while mediating the downregulation of IL2 expression via interaction with the transcription factor YY1 [60]. EZH2 is also involved in repressing STING and upstream IFN signalling, which results in diminished T cell recruitment [61]. The enzyme is also induced upon Treg activation, functioning as an epigenetic switch necessary to maintain Treg stability and their immunosuppressive phenotype [62]. Therefore, histone demethylase and methyl transferase inhibitors (e.g., KDM1A, KDM4, EZH2) are under preclinical investigation in TNBC to restore the expression of tumour suppressors and boost immunotherapy responses [63].

#### 3.2.2. Non-Coding RNAs and RNA Methylation

Multiple microRNAs (miRNAs) have been identified as mediators of immune suppression, by silencing antigen presentation genes (MHC-I, β2-microglobulin), and chemokine pathways (CXCL9/10/11) needed for T-cell recruitment, and IFN signalling [64]. Through their activity, miRNAs can exert either an oncogenic or a tumour suppressive function. For instance, the oncogenic miRNAs miR-2 and miR-10b drive EMT and upregulate the inhibitory molecules CD47 and CD24, which prevent macrophage phagocytosis and NK cell activity [65]. Similarly, elevated miR-148a expression promotes the survival of immature B cells by inhibiting the expression of the immune- inhibitory factor GADD45α and PTEN, thereby facilitating immune evasion [66]. Through TGFβ signaling, cancer cells induce the expression of miR-182 in macrophages, inducing their polarization, and its inhibition blocks TNBC progression [67]. MiR-200C also mediates polarization of TAMs into M2 by upregulation of plasminogen activator inhibitor-2 (PAI-2) and increased secretion of IL-10 [68].

On the other hand, immune suppressive miRNAs are found expressed at low levels in TNBC. Some of these miRNAs function by regulating inhibitory receptors in CD8^+^ T-cells. For instance, miR-5119 is downregulated in dendritic cells and modulates immune checkpoint ligands, including PD-L1 and indoleamine 2,3-dioxygenase 2 (IDO2) [69]. Gain of function of other miRNAs can restore immune function. Indeed, expression of miR-149-3p has been shown to reverse CD8^+^ T-cell exhaustion and increase cytokine production [70]. Similarly, expression of miR-138-5p can mitigate CD8^+^ T-cell exhaustion by downregulating PD-L1 expression in cancer cells [71]. MiR-424-5p and miR-142–5p can also regulate immune responses mediated by CD8^+^ T cells by targeting PD-L1 and the downstream PI3K/AKT signalling pathway [72,73].

Together with miRNAs, long non-coding RNAs (lncRNAs) are also involved in the regulation of immune evasion by modulating the function of monocytes, macrophages, neutrophils, and lymphocytes [74]. Among lncRNAs, LINC00514 has been shown to induce the polarization of TAMs toward an M2 anti-inflammatory phenotype [75]. Other lncRNAs are involved in the regulation of PD-L1. For instance, KRT19P3 suppresses PD-L1 expression and enhances CD8^+^ T-cell-mediated anti-tumor immunity [76]. The lncRNA BM466146 is instead involved in the upregulation of the chemokine CXCL13 in the TME, therefore promoting CD8^+^ T-cell infiltration through chemotaxis stimulated by CXCR5. In addition, CD8^+^ T cells bound to CXCL13 can recognize tumor antigens and activate cytotoxic pathways and immune response [77]. LncRNAs also affect the function of NK cells. The lncRNA MALAT1 is upregulated in TNBC and is linked to immunosuppression by reducing the effect of NK cells through inhibition of activating ligands such as MICA/B and induction of immune checkpoints [78].

Posttranscriptional regulation of immune function also involves changes in RNA methylation, with members of the m6A methyltransferase complex playing an important role. While highly expressed in TNBC cancer cells and driving tumor progression, Wilms’ tumor 1-associating protein WTAP has an inhibitory role in CD8+T cells, reducing the expression of inhibitory receptors and driving an exhausted state. However, in dendritic cells, it exerts a pro-immune effect by enhancing MHC expression and antigen presentation [79]. The m6A methyltransferase-like 3 METTL3 also enhances PD-L1 expression in TNBC cells through an m6A-IGF2BP3-dependent mechanism, affecting the efficacy of tumor immunotherapy [80]. Finally, downregulation of METTL14 in macrophages can drive CD8 + T cell activation [81].

## 4. Harnessing Immune Responses for Therapy

### 4.1. Epigenetics and Viral Mimicry

Modulation of epigenetic modifications can be used to restore tumor immune response. To this end, epigenetic regulation of transposable elements can be used to harness the organism’s intrinsic immune response to the activation of endogenous viral sequences, a response known as “viral mimicry”. Viral mimicry is an epigenetically driven process in which tumors reactivate silenced endogenous retroviruses (ERVs) and transposable elements (SINE, LINE, Alu), producing double-stranded RNA (dsRNA) or cDNA produced by reverse transcription that mimics viral infection and triggers innate immune responses [82]. The use of epigenetic therapies that exploit this mechanism is currently under extensive investigation, especially in tumors with an immune-cold environment, including some subtypes of TNBC. Due to their ability to transpose, transposable elements (TEs) can threaten genomic integrity and cellular homeostasis. However, in physiological conditions, cells use epigenetic mechanisms to silence TEs and mitigate the adverse effects they mediate. These include DNA methylation, histone modifications, and PIWI-interacting RNAs (piRNAs). In somatic cells, most TEs are permanently silenced by DNA methylation, but their activation is required for early embryonic development and cell fate determination [83,84]. Despite these controlled silencing mechanisms, several TEs can be activated during tumorigenesis via global DNA hypomethylation. Histone modifications also affect the activation of TEs, as a lack of H4K20me3, a known hallmark of cancer, is associated with TEs hypomethylation [85]. Other TEs’ repressive marks are also lost, most notably H3K9me3 and H3K27me3, which are catalyzed by the methyltransferases SETDB1 and EZH2, together with recruitment of the transcriptional repressor TRIM28 [86]. Although TEs reactivation can cause genomic instability through insertional mutagenesis and loss of regulatory elements, recent research has reported that this can bring an advantage for immune-cold cancers as it can induce anti-tumor immune responses. Through the viral mimicry response, the formation of dsRNA and cytoplasmic dsDNA generated by reverse transcription of TEs is recognized by pattern recognition receptors, such as MDA5, RIG1, TLR3, OAS, PKR, and cGAS. This binding activates a downstream signaling by recruitment of TRIF, MAVS, and STING1 and driving activation of NF-κb and transcription of type I and III IFN and chemokines, which stimulate recruitment and activation of T cells [87]. However, tumors can adapt to this response and escape viral mimicry by modulating repressive histone modifications at hypomethylated TE regions, inducing RNA degradation and editing, and downregulating dsRNA-sensing proteins [88]. For instance, cancer cells compensate for the accumulation of cytosolic dsDNA by stimulating DNA damage repair mechanisms and preventing the cGAS-STING signaling [89]. The cGAS-STING pathway is also involved in adaptation to inflammation by converting high type I IFN signaling to chronic and indolent levels, which can stimulate EMT, cancer stemness, and T cell exhaustion [90]. Therefore, therapeutic approaches that induce robust and acute IFN signaling are required to induce immune activation via this mechanism. To this end, preclinical and clinical outcomes of epigenetic studies have highlighted the promising potential of TEs activation to drive anti-tumor responses by inducing viral mimicry pharmacologically. Treatment of cancer cells with DNMTi can boost the immune system by activating type I and III IFN signaling pathways in response to dsRNA derived from ERV activation [91]. Inhibition of the H3K4me1/2 demethylase LSD1 (KDM1A) and the H3K9 methyltransferase SETDB1 has also been found to cause viral mimicry [88]. Viral mimicry and decreased expression of the dsRNA-degrading RISC complex were observed in TNBC cell lines after treatment with a catalytic LSD1 inhibitor [92]. In addition, dsRNA expression and viral mimicry can be induced in TNBC by the inhibition of EZH2 and PRMT [93,94].

**Figure 3:**
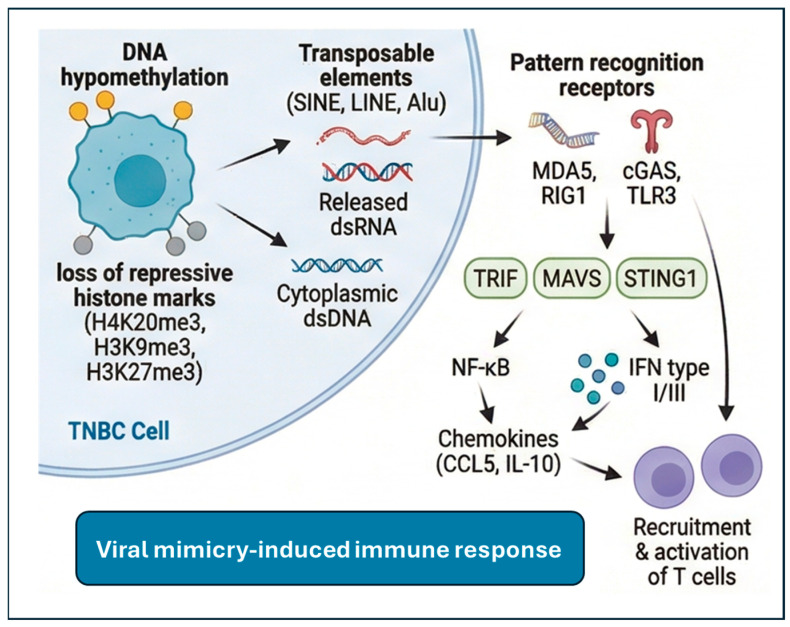
Viral mimicry responses in TNBC can be caused by inducing activation of endogenous TEs, which produce double-stranded RNA, and then trigger an interferon-driven antiviral program that reduces tumor growth and induces an immune response.

Although the therapeutic induction of viral mimicry represents a promising approach to reverse cancer immune evasion, the success of this approach may be context-dependent on the specific epigenetic landscape of tumors. The inherent heterogeneity of tumors may limit the effectiveness of these therapeutic strategies; the identification of suitable epigenetic biomarkers that can be used to stratify patients who could benefit from these therapeutic approaches is critical to ensure positive outcomes [82] (Figure 3).

### 4.2. Epigenetic Therapies

Several studies have also shown that the modulation of epigenetic modifications for reactivation of TEs can be leveraged for combined therapeutic applications. The reactivation of immune-related genes and TEs through viral mimicry to induce immune signaling in combination with checkpoint inhibitors is currently being tested for several cancers, including breast, ovarian, kidney cancers, and melanoma [95]. For instance, the combination of DNMTi with chemotherapy is being tested in clinical trials. Similarly, the use of HDAC inhibitors (HDACi) can improve the response to checkpoint inhibitors by restoring the expression of PD-L1 and inhibiting the activity of Tregs and MDSCs [96]. For instance, the combination of Entinostat with CTLA and PD-1 antibodies can block the growth and spread of TNBC cells in a syngeneic mouse model via suppression of MDSCs [97]. In addition, inhibition of BET proteins can enhance the effect of radiotherapy and induce immune responses in a syngeneic mouse model of TNBC [98]. Modulation of histone methylation has also been shown to be effective in enhancing immune responses. Inhibition of EZH2 can effectively disrupt the immune repressive function of Tregs and enhance the effect of checkpoint inhibitors [99,100,101]. In addition, blocking EZH2 in TNBC can induce the reprogramming of basal cells to a luminal phenotype by activating the expression of GATA3 and inducing a response to endocrine therapy [102]. It can also increase the expression of MHC-I by reducing H3K27me3 occupancy at the MHC-I promoter and increasing the infiltration of CD3+ T cells, as well as sensitization to paclitaxel in a syngeneic animal model of TNBC [4]. Targeting the histone demethylase LSD1 can also restore the expression of chemokines and PD-L1, as well as increasing the immune response when used with an anti-PD-1 antibody [59,103]. Furthermore, viral mimicry can be promoted by inhibitors of cell cycle kinases (CDKi), as it has been shown that CDK4-CDK6 inhibitors suppress the methyltransferase DNMT1, thus inducing viral mimicry and type III IFN response with inhibition of Tregs [104]. Therefore, based on preclinical studies using cell culture and mouse models, the combination of epigenetic drugs with current targeted therapies represents a promising approach for the treatment of TNBC, especially for those molecular subtypes that are characterized by immune-deserted tumors. Epigenetic therapies in TNBC are supported by a strong mechanistic rationale and compelling preclinical data. While single-agent activity of DNMTi and HDACi has been modest [55], this likely reflects the marked genomic and microenvironmental heterogeneity of TNBC rather than a limitation of the therapeutic concept itself. In contrast to hematological malignancies, where epigenetic dysregulation often represents a dominant driver, TNBC encompasses diverse molecular and immune phenotypes that may require subtype-specific epigenetic targeting. Toxicity and optimal combination with immunotherapy also remain important considerations, and the absence of validated predictive biomarkers currently limits precise patient selection. Nevertheless, advances in defining immune phenotypes, chromatin states, and viral mimicry signatures offer opportunities to refine therapeutic strategies. A deeper understanding of TNBC-specific epigenetic architecture through analysis of tumor and TME transcriptomic and epi-transcriptomic data may enable more effective combination approaches, positioning precision epigenetic–immunotherapy as a promising future direction in TNBC management.

## 5. Conclusions

Recent insights into TNBC heterogeneity highlight the central role of epigenetic mechanisms regulating the TME. Epigenetic alterations shape the immune landscape of TNBC by modulating immune cell activity, cytokine signaling, and immune checkpoint expression, collectively contributing to immune evasion. This growing body of evidence has informed the development of epigenetic modulating drugs as anti-cancer therapy, and several ongoing clinical trials are evaluating these agents in combination therapies. These focus on enhancing the effect of chemotherapy and immunotherapy using checkpoint inhibitors (Table 1). This approach, together with the induction of viral mimicry responses through reactivation of endogenous retroviral sequences and transposons, represents a new strategy for the development of targeted therapies for TNBC. Given the marked molecular and immune heterogeneity of TNBC, a precision epigenetic–immunotherapy framework tailored to distinct immune phenotypes and TNBC molecular subtypes may be needed to optimize patient selection and maximize therapeutic benefit. Such an approach may enable rational integration of epigenetic therapies with immunomodulatory strategies, ultimately advancing personalized treatment for TNBC.

## Figures and Tables

**Table 1:** Currently open clinical trials for TNBC using combination epigenetic therapy.

Trial Number	Phase	Drugs	End
NCT07351487	Phase II	Sintilimab and Bevacizumab	2028
NCT02393794	Phase I/II	Cisplatin + Romidepsin + Nivolumab	2026
NCT04192903	Phase II	Chidamide + Cisplatin	ND *
NCT04315233	Phase I	Ribociclib + Belinostat	2026
NCT02957968	Phase II	Decitabine + Paclitaxel/Doxorubicin/Cyclophosphamide/Carboplatin + Pembrolizumab	2026
NCT05673200	Phase II	Cedazuridine + Decitabine + Paclitaxel+ Pembrolizumab	2027
NCT03805399	Phase I/II	Pyrotinib + Capecitabine/Everolimus + SHR3680 + SHR6390 + SHR2554/SHR1210 + Paclitaxel	ND
NCT05327010	Phase II	ZEN003694 + Talazoparib	2026
NCT05422794	Phase I	ZEN003694 + Pembrolizumab + Paclitaxel	2027
NCT05372640	Phase I	ZEN003694 + Abemaciclib	2026
NCT06371807	Phase II	Pembrolizumab + Paclixaxel + Carboplatin	2026

* ND = not determined.

## Data Availability

No new data were created or analyzed in this study. Data sharing is not applicable.

## Abbreviations

The following abbreviations are used in this manuscript:

TNBC	Triple negative breast cancer
BL	Basal-like
LAR	Luminal androgen receptor
M	Mesenchymal
EMT	Epithelial-to-mesenchymal transition
TME	Tumor microenvironment
CSC	Cancer stem cell
TILs	Tumor infiltrating lymphocytes
NK	Natural killer
TAMs	Tumor-associated macrophages
Tregs	Regulatory T cells
MDSCs	Myeloid-derived suppressor cells
MHC	Major histocompatibility complex
HLA	Human leukocyte antigen
IFN	Interferon
ICIs	Immune checkpoint inhibitors
DMNT	DNA methyltransferase
HAT	Histone acetyltransferase
KDM	Histone demethylase
DNMTi	DNA methyltransferase inhibitors
HDACi	Histone deacetylase inhibitors
CDKi	Cyclin-dependent kinase inhibitors
miRNA	MicroRNA
lncRNA	Long non-coding RNA
dsRNA	Double stranded RNA
piRNA	PIWI-interacting RNA
